# A case report: an unusual case of ulcerative intestinal IgG4-related disease with clinical features overlapping with probable intestinal Behcet’s disease

**DOI:** 10.1186/s12876-026-05344-w

**Published:** 2026-09-25

**Authors:** Shukai Zhan, Jing Yang, Wenlong Liang, Hongli Huang, Yongjian Zhou

**Affiliations:** 1 Department of Gastroenterology and Hepatology, Guangzhou First People’s Hospital, Guangzhou Medical University, No.1, Pan Fu Road, Guangzhou, 510180 P.R. China; 2 Department of Pathology, Guangzhou First People’s Hospital, Guangzhou Medical University, Guangzhou, PR China; 3 Department of Gastrointestinal Surgery, Guangzhou First People’s Hospital, Guangzhou Medical University, Guangzhou, PR China

**Keywords:** IgG4-related disease, Intestinal Behcet’s disease, Ulceration, Overlap, Case report

## Abstract

**Background:**

IgG4-related disease (IgG4-RD) is a systemic fibroinflammatory condition characterized by IgG4-positive plasma cell infiltration, typically presenting with organ enlargement or tumor-like masses. Luminal gastrointestinal involvement is rare, and ulcerative lesions are even less common. Overlap syndromes of IgG4-RD with other immune disorders have been reported, but overlap of its clinical features with those of intestinal Behcet’s disease has not been documented.

**Case presentation:**

A 62-year-old man with a history of recurrent oral ulcers presented with sudden hematochezia and hypovolemic shock. Imaging revealed sclerosing mesenteritis, and endoscopy identified multiple small intestinal ulcers. Histopathological examination of the resected intestinal segment demonstrated dense infiltration of IgG4-positive plasma cells (70/HPF, IgG4⁺/IgG⁺ ratio 42.3%), confirming intestinal IgG4-RD. The patient also met diagnostic criteria for probable intestinal Behcet’s disease based on recurrent oral ulcers, crateriform intestinal ulcers, and histologic evidence of vasculitis. Following partial enterectomy and postoperative immunosuppressive therapy (prednisone and cyclophosphamide), the patient achieved sustained remission with no recurrence during the one-year follow-up.

**Conclusions:**

This is the first reported case of concurrent intestinal IgG4-RD and probable intestinal Behcet’s disease. The overlapping presentation expands the clinical spectrum of IgG4-RD and suggests a potential pathogenic link between these two immune-mediated conditions.

**Supplementary Information:**

The online version contains supplementary material available at https://doi.org/10.1186/s12876-026-05344-w.

## Background

IgG4 related disease (IgG4-RD) is a systemic autoimmune disease characterized by infiltration of IgG4-positive plasma cells into organs and/or elevated serum IgG4 concentrations. The pancreas, salivary glands, lymph nodes and bile ducts are frequently affected, mainly manifesting as tumefactive and fibrotic lesions. Therefore, it is always misdiagnosed as a malignant tumor [1, 2]. Although IgG4-RD can theoretically affect all organs, gastrointestinal tract involvement is relatively rare and has only been reported occasionally [3]. Previous reports have shown that gastrointestinal IgG4-RD presents with diverse clinical manifestations, mostly the aforementioned tumefactive lesions such as wall thickening, submucosal tumors, nodules or polyps [4], whereas ulceration is rare, particularly in the small intestine.

In addition, overlap syndromes of IgG4-RD with other diseases have been reported in some cases, such as anti-neutrophil cytoplasmic antibody (ANCA) associated vasculitis, Graves’ disease and inflammatory bowel disease (IBD) [5–7]. However, to our knowledge, the coexistence of intestinal IgG4-RD with the other disease have never been reported.

We present a case of IgG4-RD manifesting as rare multiple small intestinal ulceration and sclerosing mesenteritis, with clinical features concurrently suggestive of probable intestinal Behcet’s disease. To our knowledge, this is the first documented case of concurrent intestinal IgG4-RD and probable intestinal Behcet’s disease.

## Case presentation

A 62-year-old man was admitted to hospital with sudden hematochezia lasting for 6 h. Six hours before admission, he experienced mild abdominal pain without obvious trigger, followed by four episodes of hematochezia. The total volume of bloody stools was nearly 6000 ml, and he lost consciousness for several minutes. Physical examination revealed hypotension (88/56 mmHg) and tachycardia (112 bpm); signs of anemia, such as pale skin and mucous membranes, were also noted. The abdomen was soft, without tenderness or rebound tenderness. Hemorrhoids were visible in the anus, but no active bleeding was observed.

The patient had experienced recurrent oral ulcers for decades but not sought medical attention because the symptoms were mild. Three years earlier, he had undergone an anal fistulectomy, with no recurrence since then. He had no history of tobacco or alcohol use, or long-term medication, and denied any history of food or drug allergies. He also denied any family history of malignant tumors or infectious diseases.

A complete blood count revealed a white-cell count of 11.82 × 10^9^/L (reference range 3.5 × 10^9^ ~ 9.5 × 10^9^), an absolute lymphocyte count of 6.95 × 10^9^/L (reference range 1 × 10^9^/L ~ 3.3 × 10^9^/L). The plasma C-reactive protein level was 12.22 mg/L (reference range < 5 mg/L). In addition, the interferon-γ release assay result for tuberculosis was positive. The blood levels of electrolytes, cytokines, autoantibodies (including ANA, ASCA, c-ANCA and p-ANCA), procalcitonin and IgG4 were normal. No abnormalities were found on chest computed tomography (CT), kidney function, liver function, and coagulation (Supplementary Table 1).

**Fig. 1:**
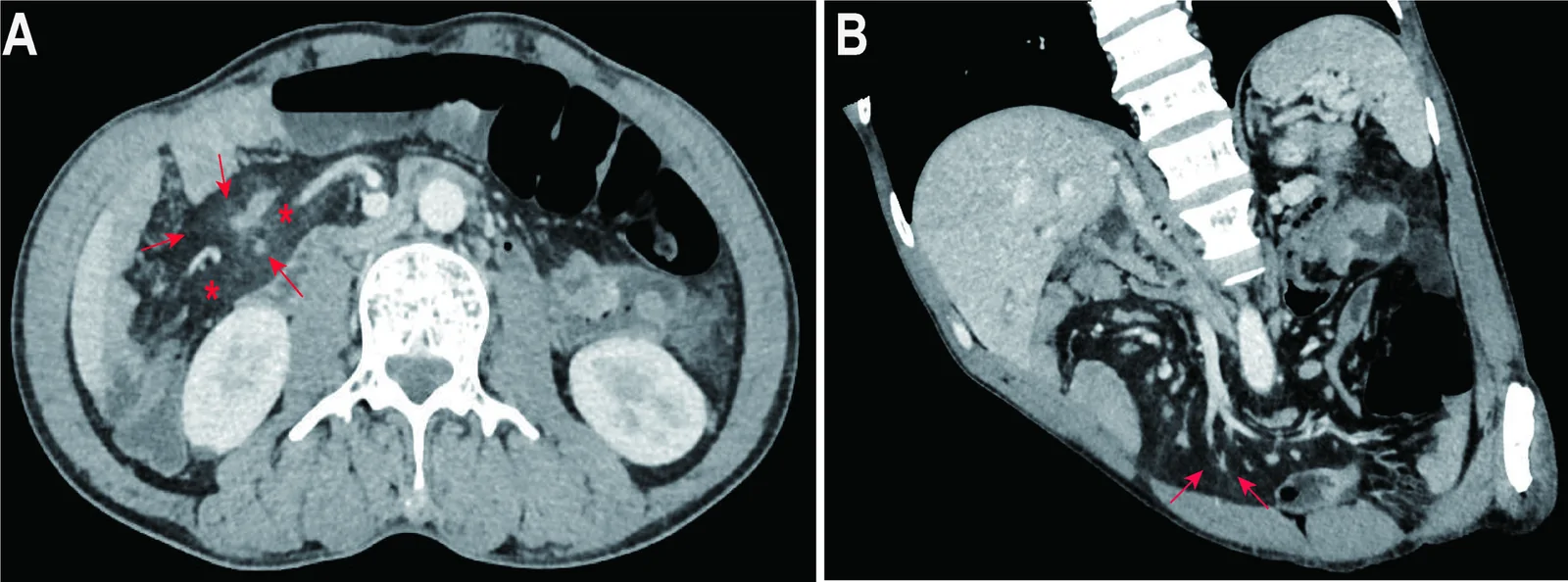
Abdominal computed tomography **A** The density of the mesentery was abnormally high (asterisks) and the mesenteric fat contained multiple soft tissue nodules (arrows). **B** Hypoattenuating fat surrounded the mesenteric vessels

**Fig. 2:**
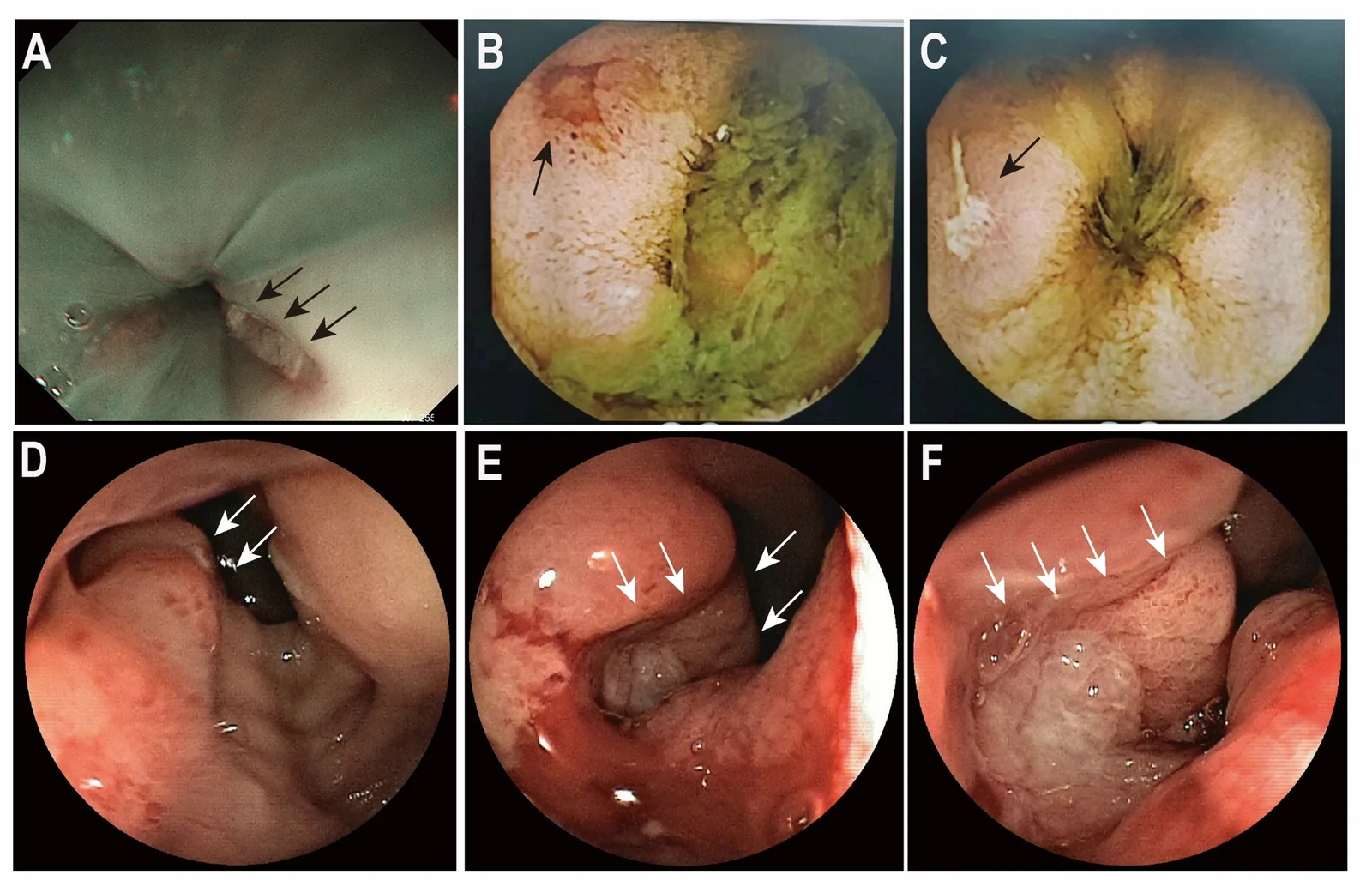
Gastrointestinal endoscopy **A** Gastroscopy with narrow band imaging detected an ulcer in the lower esophagus. **B**, **C** Capsule endoscopy revealed several ulcerations in the ileum. **D**, **E**, **F** Double-balloon small bowel endoscopy identified a swollen lesion with superficial ulceration in the ileum. Images were taken from different distances

Abdominal CT showed abnormally high density of the mesentery (Fig. 1A). In addition, multiple soft tissue nodules were found in the high-density mesentery (Fig. 1A), and the mesenteric vessels were surrounded by hypoattenuating fat (Fig. 1B). Gastroscopy revealed an ulcer in the esophagus (Fig. 2A). Capsule endoscopy revealed scattered ulcerations in the ileum (Fig. 2B, C). To further characterize the findings, we performed double-balloon small bowel endoscopy, which identified a swelling lesion with superficial ulceration eighty centimeters from the ileocecal valve, causing luminal stenosis; no bleeding site was detected (Fig. 2D, E, F).

**Fig. 3:**
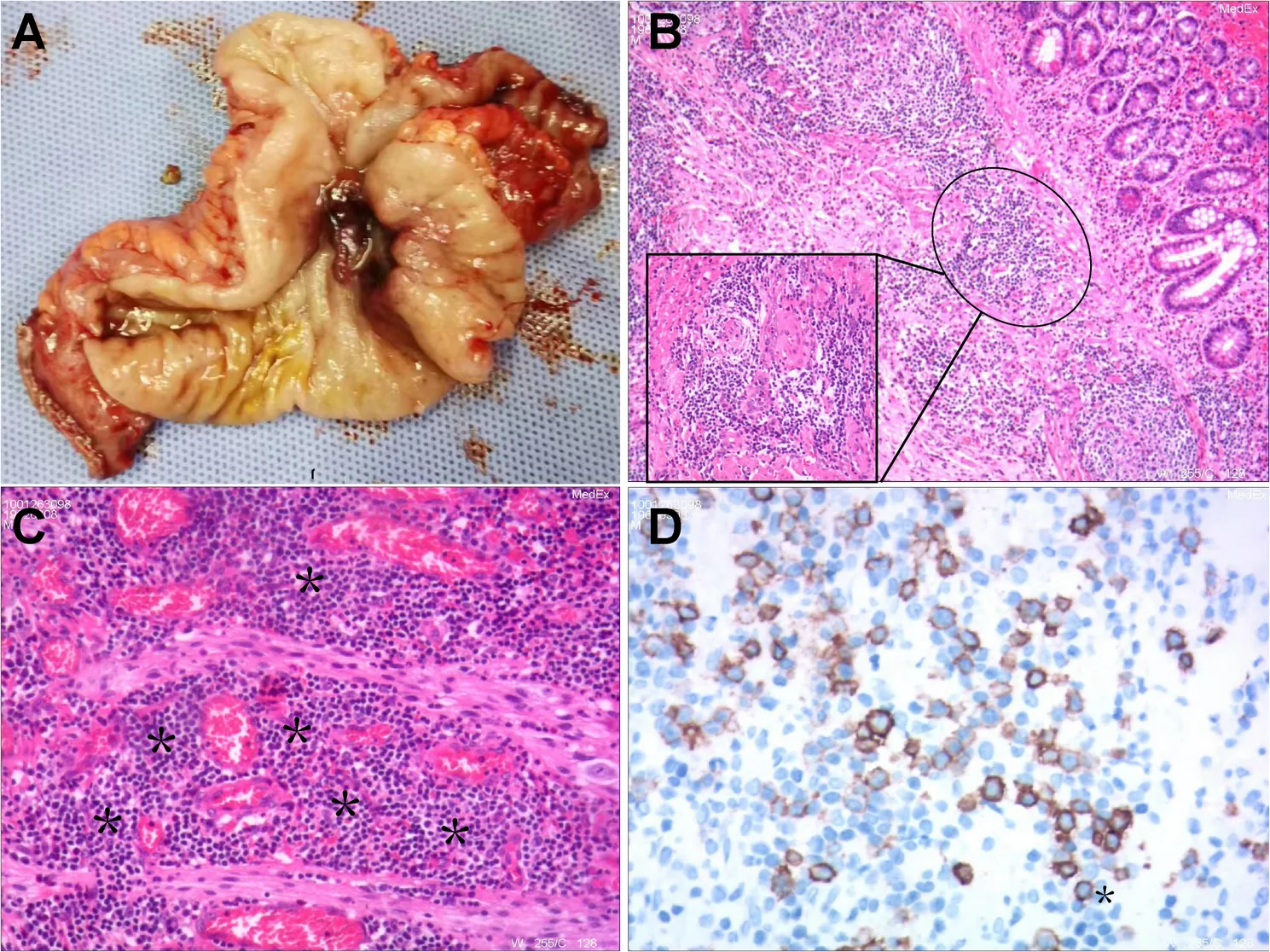
Pathological specimen **A** Resected intestinal segment showed two crateriform ulcers (2.3 × 1.8 × 0.5 cm and 0.6 × 0.6 × 0.2 cm) with surrounding mucosal swelling. **B** Interstitial fibrous tissue accumulated in extracellular matrix. In some vessels, aggregated lymphocytes, along with fibrous tissue (circle), caused wall destruction and luminal obliteration. **C** Aggregated lymphocytes (asterisks) transmurally infiltrated the vessel walls. **D** Immunohistochemistry indicated IgG4-positive plasma cells mainly infiltrated the intestinal submucosa, muscular layer, and serosa, accounting for 42.3% of IgG-positive plasma cells.

The patient received a proton pump inhibitor and fluid resuscitation. Given the emergent presentation and the unknown etiology, somatostatin was administered empirically based on its well-established efficacy in controlling various types of hemorrhage. After that, his symptoms improved. However, two weeks after admission, the patient’s hematochezia recurred. Following a multidisciplinary team consultation and based on the examination results, a partial enterectomy was recommended. Two crateriform ulcers were seen in the resected segment with surrounding mucosal swelling (Fig. 3A). Microscopy revealed the decreased intestinal crypts and accumulation of extracellular interstitial fibrous tissue (Fig. 3B). In addition, aggregated lymphocytes, along with fibrous tissue, transmurally infiltrated the vessel walls and caused wall destruction, with luminal obliteration seen in some vessels (Fig. 3B, C). Immunohistochemistry indicated an exceptionally large number of IgG4-positive plasma cells, approximately 70/HPF, and the ratio of IgG4^+^/IgG^+^ cells was 42.3% (Fig. 3D).

**Fig. 4:**
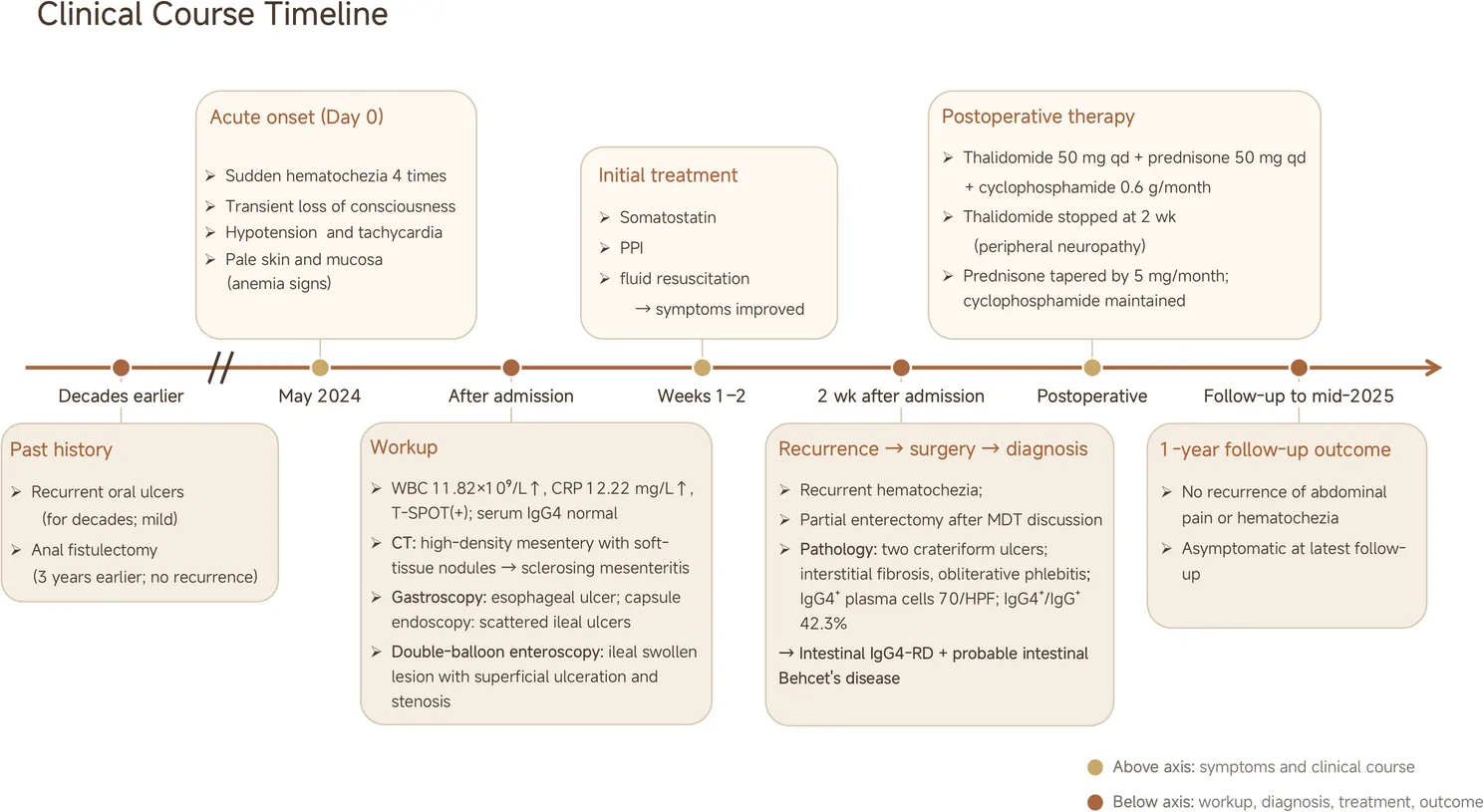
Clinical timeline of the patient according to CARE guidelines. The timeline summarizes the patient’s clinical course from the onset of recurrent oral ulcers to one-year post-diagnosis. Above axis indicated his symptoms and clinical course, below axis represented the diagnosis, treatment and the outcome

Postoperatively, after tuberculosis was excluded based on normal chest CT findings and no respiratory symptoms, the patient received thalidomide 50 mg once a day, prednisone 50 mg once a day, and cyclophosphamide 0.6 g once a month. Two weeks later, thalidomide was discontinued because of peripheral neuropathy. After that, cyclophosphamide and prednisone were sustained (Prednisone sequentially decreased by 5 mg every month). After a year of treatment, no gastrointestinal hemorrhage had recurred, and the patient recovered well postoperatively. As of the latest follow-up, the patient remains asymptomatic, with no recurrence of abdominal pain and hematochezia (The complete clinical timeline is summarized in Fig. 4).

## Discussion and conclusions

IgG4-RD was first recognized over twenty years ago, initially manifesting as sclerosing pancreatitis [8]. Its histological hallmarks included dense lymphoplasmacytic infiltration of IgG4-positive plasma cells, obliterative phlebitis and storiform fibrosis [9]. Although IgG4-RD can theoretically affect any organ as a systemic fibroinflammatory disorder, luminal gastrointestinal involvement is rare, and small intestinal ulceration is even less common [10].

In our patient, the small intestine and mesentery were involved. While the pancreas or biliary ducts, the organs most commonly affected, were unremarkable. The diagnosis of IgG4-RD was established base on *European Guideline on IgG4-relatedc digestive disease – UEG and SGF evidence-based recommendations (2020)* and *The 2019 American College of Rheumatology/European League Against Rheumatism classification criteria for IgG4-related disease* [3, 11]. The 2020 European requires more than 50 IgG4-positive cells per HPF and an IgG4^+^/IgG^+^ ratio above 40% for the histopathological diagnosis of intestinal IgG4-RD. Our patient’s resected intestinal specimen demonstrated 70 IgG4-positive plasma cells per HPF and IgG4^+^/IgG^+^ ratio of 42.3%, which unequivocally exceeds the cut-off. Under the 2019 classification criteria, obliterative phlebitis and dense IgG4^+^ plasma cell infiltration fulfill the histopathological domain.

Although classic “storiform fibrosis” was not prominent and the serum IgG4 level was normal, neither finding excludes the diagnosis: serum IgG4 was rarely elevated in the cases of bowel involvement and is normal at diagnosis in up to 50% of patients [12]. The combination of dense IgG4^+^ plasma cell infiltration, a high IgG4^+^/IgG^+^ ratio, and the presence of obliterative phlebitis provided robust evidence for the diagnosis.

The differential diagnosis of intestinal IgG4-RD is particularly challenging. Several important mimickers were carefully considered in our patient. Crohn’s disease was a primary consideration, given the oral ulcers, prior anal fistulectomy, and stenosing small-intestinal ulcers. However, the absence of non-caseating granulomas, and transmural lymphoid aggregates, the isolated ileal lesion without typical “skip lesions”, cobblestone-like appearance or fissuring ulcers, and negative ASCA or ANCA serology argue against Crohn’s disease. Although intestinal IgG4-postive plasma cell infiltration has been proposed as a possible IBD subtype rather than systemic IgG4-RD, the presence of obliterative phlebitis – a hallmark of IgG4-RD – and the exceptionally high density of IgG4^+^ cells strongly favor a primary IgG4-RD diagnosis rather than an IgG4-rich IBD subtype in our patient [7].

Intestinal tuberculosis was also considered, given the positive interferon-γ release assay. However, chest CT showed no evidence of active or latent pulmonary tuberculosis, and pathology revealed no caseating granulomas or acid-fast bacilli. Malignancy was excluded by the absence of atypical cells or mass lesions. Reactive IgG4^+^ infiltration secondary to chronic inflammation yields lower densities and ratios. While in our patient, the extensive distribution of IgG4^+^ cells and high ratio of IgG4^+^/IgG^+^ strongly support a primary IgG4-RD.

Notably, our patient also met the criteria for probable intestinal Behcet’s disease according to the Korean diagnostic criteria proposed by Cheon et al. (2009) [13]. According to the guideline, patient with typical intestinal ulcers and some systemic Behcet’s disease symptoms (but not fully meet the criteria of systemic Behcet’s disease) are classified as the probable type. Our patient had typical crateriform ulcers with elevated margins in the ileum, while recurrent oral aphthous ulceration was the only systemic feature and did not fulfill the criteria for systemic Behcet’s disease. Therefore, a diagnosis of probable intestinal Behcet’s disease is appropriate.

The pathogenesis underlying the overlapping features of IgG4-RD with other immune-mediated conditions remains unclear. The prevailing hypothesis suggested it was the interconnected immune dysregulation that drove this phenomenon. For example, of the aberrant B-cell response in IgG4-RD may elevate ANCA levels and predispose to vasculitis. Immune hyperactivity from other diseases may reciprocally drive IgG4-differentiation of the B cell [5]. Similarly, the abnormal B-cell response in our patient may triggered helper T-cell proliferation and pro-inflammatory cytokines release (IFN-γ, TNF-α, IL-17, IL-12), culminating in Behcet’s-like features [14]. Notably, ulcerative presentation contrasts with the organomegaly typical of IgG4-RD [15]. Ulcer formation is likely multifactorial: both the IgG4-related fibroinflammatory process and the Behcet’s-associated vasculitis could contribute to tissue breakdown and ulceration, although the precise contribution of each disease process remains speculative.

Both intestinal Behcet’s disease and IgG4-RD are immune-mediated disorders, and suppression of inflammatory exacerbations is the common therapeutic target. Glucocorticoids and immunosuppressive agents, such as azathioprine, cyclophosphamide, thalidomide, and leflunomide, were commonly used. In our patient, prednisone served as first-line induction therapy for both IgG4-RD and intestinal Behcet’s disease. Cyclophosphamide was added as a steroid-sparing agent, which is a recognized therapy for severe cases of both conditions, particularly when a vasculitis is present. Thalidomide was initially chosen for its efficacy against intestinal ulcers in Behcet’s disease but was discontinued due to peripheral neuropathy. Biological agents showed some efficacy in refractory or steroid-dependent cases [16].

A comprehensive literature review (PubMed and Web of Science, up to December 2025; terms like “IgG4-related disease” AND “Behcet’s disease” AND “intestine”) identified only one case of sequential involvement, in which a patient was initially diagnosed with Behcet’s disease and fifteen years later presented with acute kidney injury and elevated serum IgG4 levels [17]. To our knowledge, no case of simultaneous intestinal ulcerative IgG4-RD with clinical features overlapping with intestinal Behcet’s disease has been documented. Our case therefore represents the first reported instance of this unusual presentation, expanding the known clinical spectrum of intestinal IgG4-RD.

Several limitations exist. First, as a single-case report, no causal inference can be made. Second, the diagnosis of intestinal Behcet’s disease remains “probable” rather than definitive, as HLA-B51 and pathergy tests were not performed. Third, we cannot completely exclude an IgG4-rich subtype of inflammatory bowel disease, although the histopathological findings strongly favor primary IgG4-RD. Fourth, the follow-up period is relatively short for two potentially relapsing-remitting conditions, and longer-term surveillance is warranted.

We report a rare case of intestinal ulceration presenting with sudden hematochezia, ultimately diagnosed as intestinal IgG4-RD with clinical features overlapping with probable intestinal Behcet’s disease. While the underlying mechanisms remain unclear, our case suggests a potential pathogenic link between IgG4-RD and Behcet’s disease, warranting further investigation.

**Figure legends**

Figure 1. Abdominal computed tomography. **A** Density of mesentery was abnormally high (asterisks) and the mesenteric fat contained multiple soft tissue nodes (arrows) in computed tomography. **B** Hypoattenuating fat surrounded the mesenteric vessels.

Figure 2. Gastrointestinal Endoscopy. **A** Gastroscopy with narrow band imaging detected an ulcer in the lower esophagus. **B**, **C** Capsule endoscopy discovered several ulcerations could be found in the ileum. **D**, **E**, **F** Double-balloon small bowel endoscopy found a swelling lesion with superficial ulceration in the ileum. It was taken photo from different distance.

Figure 3**. **Pathological specimen. **A** Resected intestinal segment showed two crateriform ulcers (2.3 × 1.8 × 0.5 cm and 0.6 × 0.6 × 0.2 cm) with surrounding mucosal swelling. **B** In some vessels, aggregated lymphocytes, along with fibrous tissue (circle), caused wall destruction and luminal obliteration. **C** Aggregated lymphocytes (asterisks) transmurally infiltrated the vessel walls. **D** Immunohistochemistry indicated IgG4-positive plasma cells mainly infiltrated the intestinal submucosa, muscular layer, and serosa, accounting for 42.3% of IgG-positive plasma cells.

Figure 4. Clinical timeline of the patient according to CARE guidelines. The timeline summarizes the patient’s clinical course from the onset of recurrent oral ulcers to one-year post-diagnosis. Above axis indicated his symptoms and clinical course, below axis represented the diagnosis, treatment and the outcome.

## Supplementary information

Below is the link to the electronic supplementary material.

Supplementary Material 1

Supplementary Material 2

## Data Availability

No datasets were generated or analysed during the current study.

## **Abbreviations**

IgG4-RD: IgG4-related disease
ANCA: Anti-neutrophil cytoplasmic antibody
IBD: Inflammatory bowel disease
CT: Computed tomography
