# Supplementary material for: A case report: an unusual case of ulcerative intestinal IgG4-related disease with clinical features overlapping with probable intestinal Behcet’s disease
Source: BMC Gastroenterol. 2026 Sep 25;26:589. doi: 10.1186/s12876-026-05344-w (PMC13621652; doi:10.1186/s12876-026-05344-w)
Supplement: Supplementary file 1 [file 12876_2026_5344_MOESM2_ESM.pdf]

CARE case report guidelines

CARE Checklist of information to include when writing a case report

(cc) BY-NC-ND

Topic	Item	Checklist item description	Reported on Line
Title	1	The diagnosis or intervention of primary focus followed by the words "case report"	Line 1
Key Words	2	2 to 5 key words that identify diagnoses or interventions in this case report, including "case report"	Line 49
Abstract (no references)	3a	Introduction: What is unique about this case and what does it add to the scientific literature?	Line 27 to 30
	3b	Main symptoms and/or important clinical findings	Line 32 to 38
	3c	The main diagnoses, therapeutic interventions, and outcomes	Line 36 to 37; Line 39 to 41
	3d	Conclusion—What is the main "take-away" lesson(s) from this case?	Line 44 to 46
Introduction	4	One or two paragraphs summarizing why this case is unique (**may include references**)	Line 56 to 70
Patient Information	5a	De-identified patient specific information.	Line 73
	5b	Primary concerns and symptoms of the patient.	Line 73 to 76; Line 108 to 109
	5c	Medical, family, and psycho-social history including relevant genetic information	Line 81 to 85
	5d	Relevant past interventions with outcomes	Line 81 to 82
Clinical Findings	6	Describe significant physical examination (PE) and important clinical findings.	Line 77 to 80
Timeline	7	Historical and current information from this episode of care organized as a timeline	Line 128 (Figure 4)
Diagnostic Assessment	8a	Diagnostic testing (such as PE, laboratory testing, imaging, surveys).	Line 86 to 101; Line 110 to 118
	8b	Diagnostic challenges (such as access to testing, financial, or cultural)	N/A
	8c	Diagnosis (including other diagnoses considered)	Line 137 to 179
	8d	Prognosis (such as staging in oncology) where applicable	N/A
Therapeutic Intervention	9a	Types of therapeutic intervention (such as pharmacologic, surgical, preventive, self-care)	Line 105 to 108; Line 109 to 110
	9b	Administration of therapeutic intervention (such as dosage, strength, duration)	Line 121 to 125
	9c	Changes in therapeutic intervention (with rationale)	Line 108 to 110; Line 122 to 125
Follow-up and Outcomes	10a	Clinician and patient-assessed outcomes (if available)	Line 125 to 127
	10b	Important follow-up diagnostic and other test results	N/A
	10c	Intervention adherence and tolerability (How was this assessed?)	Line 122 to 127
	10d	Adverse and unanticipated events	Line 122 to 123
Discussion	11a	A scientific discussion of the strengths AND limitations associated with this case report	Line 203 to 218
	11b	Discussion of the relevant medical literature **with references**.	Line 131 to 136; Line 154 to 171; Line 193 to 202
	11c	The scientific rationale for any conclusions (including assessment of possible causes)	Line 180 to 192
	11d	The primary "take-away" lessons of this case report (without references) in a one paragraph conclusion	Line 219 to 223
Patient Perspective	12	The patient should share their perspective in one to two paragraphs on the treatment(s) they received	Line 126 to 127
Informed Consent	13	Did the patient give informed consent? Please provide if requested	**Yes** ☑ **No** ☐
