# Supplementary material for: A case report: an unusual case of ulcerative intestinal IgG4-related disease with clinical features overlapping with probable intestinal Behcet’s disease
Source: BMC Gastroenterol. 2026 Sep 25;26:589. doi: 10.1186/s12876-026-05344-w (PMC13621652; doi:10.1186/s12876-026-05344-w)
Supplement: Supplementary file 2 [file 12876_2026_5344_MOESM1_ESM.docx]

**Supplement Table1. Laboratory Data**

| **Variable** | **Reference Range,  This Hospital^※^** | **On This  Admission** |
| --- | --- | --- |
| *White-cell count (×10^9^/L)* | *3.50 ~ 9.50* | *11.82* |
| Differential count (×10^9^/L) |  |  |
| Neutrophils | 2.0 ~ 6.9 | 3.8 |
| *Lymphocytes* | *1.00 ~ 3.30* | *6.95* |
| *Monocytes* | *0.20 ~ 0.70* | *0.74* |
| Eosinophils | 0.05 ~ 0.30 | 0.30 |
| Basophils | 0 ~ 0.20 | 0.03 |
| *Hemoglobin (g/L)* | *130 ~ 175* | *78* |
| *Hematocrit (%)* | *42 ~ 49* | *23.7* |
| Platelet count (×10^9^/L) | 125 ~ 350 | 272 |
| Sodium (mmol/L) | 137.0 ~ 147.0 | 137.6 |
| Potassium (mmol/L) | 3.5 ~ 5.3 | 4.0 |
| Chloride (mmol/L) | 99.0 ~ 110.0 | 109.7 |
| Carbon dioxide (mmol/L) | 21 ~ 31 | 24 |
| Urea nitrogen (mg/dl) | 10.08 ~ 26.61 | 25.57 |
| Creatinine (mg/dl) | 0.64 ~ 1.26 | 0.83 |
| Glucose (mmol/L) | 3.9 ~ 11.1 | 7.15 |
| Aspartate aminotransferase (U/L) | 15 ~ 40 | 21 |
| Alanine aminotransferase (U/L) | 9 ~ 50 | 14 |
| *Alkaline phosphatase (U/L)* | *45 ~ 125* | *43* |
| Total bilirubin (μmol/L) | 0 ~ 26.0 | 9.2 |
| *Albumin (g/L)* | *40.0 ~ 55.0* | *27.8* |
| *Total protein (g/L)* | *65 ~ 85* | *52.2* |
| *C-reactive protein (mg/L)* | *< 5* | *12.22* |
| *Erythrocyte sedimentation rate (mm/hr)* | *0 ~ 15* | *30* |
| Prothrombin time (sec) | 11 ~ 14.5 | 15.0 |
| Prothrombin-time international normalized ratio | 0.85 ~ 1.20 | 1.18 |
| Alpha-fetoprotein (ng/mL) | 0 ~ 7 | 1.5 |
| Carbohydrate antigen-199 (U/mL) | 0 ~ 30 | 2.32 |
| Carcinoma Embryonic Antigen (ng/mL) | 0 ~ 5 | 0.51 |
| Immunoglobulin A (g/L) | 1.0 ~ 4.2 | 3.0300 |
| Immunoglobulin E (ng/mL) | 0 ~ 240 | 688.900 |
| Immunoglobulin G (g/L) | 8.6 ~ 17.4 | 11.220 |
| Immunoglobulin G4 (g/L) | ≤2.00 | 0.28 |
| Complement C3c (g/L) | 0.7 ~ 1.4 | 0.903 |
| Complement C4 (g/L) | 0.1 ~ 0.4 | 0.227 |
| Anti-cardiolipin antibody immunoglobulin G (GPLU/mL) | 0 ~ 10 | 3.99 |
| Anti-cardiolipin antibody immunoglobulin M (MPLU/mL) | 0 ~ 10 | 2.39 |
| Anti-myeloperoxidase antibody  immunoglobulin G (Absorbance Unit/mL) | 0 ~ 20 | 6.09 |
| Anti-protease 3 antibody immunoglobulin G  (Absorbance Unit/mL) | 0 ~ 20 | < 2.00 |
| Antinuclear antibodies (Absorbance Unit/mL) | < 32 | < 4.00 |
| Anti-double-stranded DNA antibody | Negative | Negative |
| Anti-ribosomal P-protein antibody | Negative | Negative |
| Anti-SSA antibody | Negative | Negative |
| Anti-SSB antibody | Negative | Negative |
| Anti-Smith antibody | Negative | Negative |
| Anti-Jo-1 antibody | Negative | Negative |
| Anti-Scl-70 antibody | Negative | Negative |
| Anti-nucleosome antibody | Negative | Negative |
| Anti-centromere-protein-B antibody | Negative | Negative |
| Antihistone antibody | Negative | Negative |
| *Interferon-γ release assay of tuberculosis* | *Negative* | *Positive* |
| ※ Reference values are affected by many variables, including the patient population and the laboratory methods used. The ranges used at Guangzhou First People's Hospital are for adults who are not pregnant and do not have medical conditions that could affect the results. They may therefore not be appropriate for all patients. The italic mean the abnormal results. | | |
